# P-2202. Immune response after hepatitis B vaccination among healthcare providers in Uzbekistan

**DOI:** 10.1093/ofid/ofae631.2356

**Published:** 2025-01-29

**Authors:** Rafail Ibragimov, Roberta Horth, Dilyara Nabirova, Alfiya Denebayeva, Botirjon Kurbanov

**Affiliations:** Central Asia Advanced Field Epidemiology Training Program, Tashkent, Toshkent, Uzbekistan; US Centers for Disease Control and Prevention, Dulles, Virginia; CDC Central Asia office, Almaty, Almaty, Kazakhstan; Almaty City Center for Prevention and Control of AIDS, HIV Center, Kazakhstan,, Almaty, Almaty, Kazakhstan; Sanitary-Epidemiological Tranquility and Public Health Committee Tashkent, Uzbekistan, Tashkent, Toshkent, Uzbekistan

## Abstract

**Background:**

About 2.5 million people in Uzbekistan have Hepatitis B virus (HBV). Mass HBV vaccination of healthcare providers was conducted in 2015 and 2022. Post-vaccination immunity testing was never performed. We aimed to estimate post-vaccination immune response and determine associated factors.

**Table 1.**
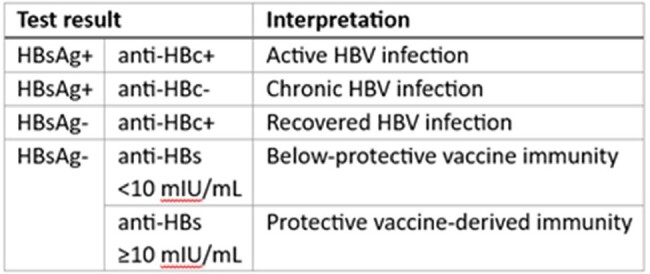
HBV tests and intepretation

**Methods:**

We conducted a cross-sectional study in Tashkent in 2023. Participants were randomly-selected from a database of providers vaccinated in the 2015 and 2022 campaigns. Interviews and blood collection for hepatitis B surface antigen, anti-HBs, and core antigen testing was performed at the workplace (Table 1). We assessed differences in mean titers using the Kruskal-Wallis test and factors associated with protective immune response (anti-HBs ≥10mIU/mL) using multivariable Poisson regression expressed as adjusted prevalence ratios (aPR).

**Table 2.**
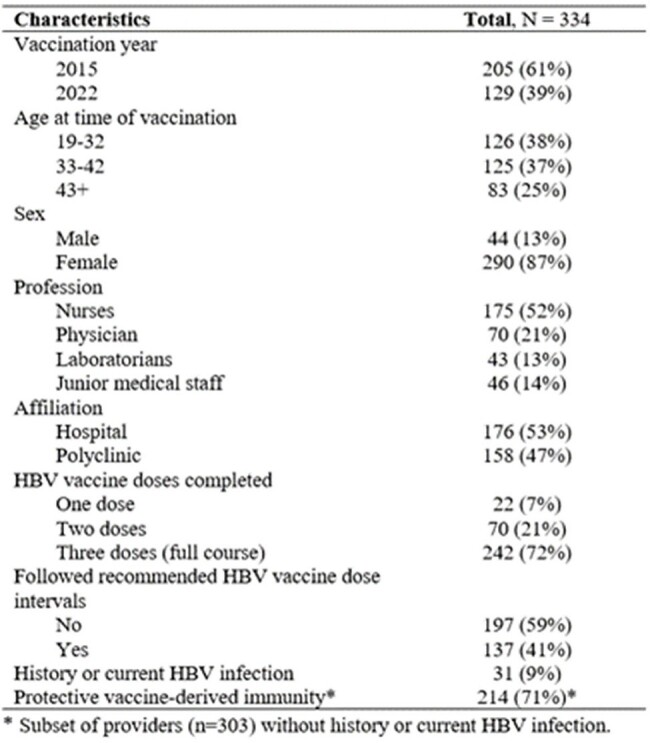
Characteristics of healthcare providers vaccinated against HBV, Uzbekistan

**Results:**

Of 334 participants, 61% were vaccinated in 2015 and 39% in 2022 (Table 2). Average age was 40 years old (interquartile range:35–49 years), 87% were female, 52% were nurses, 53% worked in hospitals, and 9% had current or past HBV infection. Among those without prior infection (n=303), 71% had protective titers. Three-quarters (72%) had completed the entire 3-dose course, 21% had 2 doses, and 7% 1 dose; 41% followed recommended intervals between doses. Mean immune titers differed by age (68 mIU/mL among 19–32-year-olds vs 31 mIU/mL among >42-year-olds, p=0.021). The proportion with protective immunity did not differ by year vaccinated (70% in 2015 vs 71% in 2022), dose number (72% for those with 3 doses vs 67% with ≤2) or dose intervals (74% followed recommendation and 69% didn’t). Nurses and laboratorians were more likely than physicians to have protective immunity (aPR=1.11, 95% confidence intervals [CI]: 1.00–1.24, p=0.042 and aPR=1.13, CI:1.04–1.22, p=0.003, respectively), and primary care workers were less likely than hospital workers to be protected (aPR=0.91, CI:0.87-0.98, p=0.009) (Figure 1).

**Figure 1.**
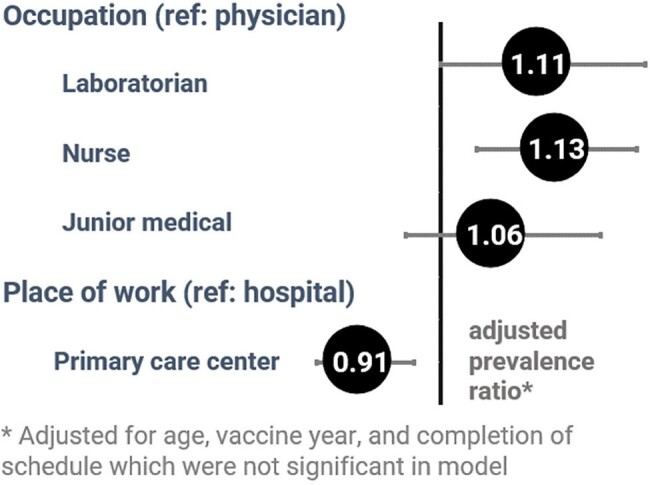
Forest plot of factors associated with protective immunity following HBV vaccination among healthcare providers in Uzbekistan

**Conclusion:**

A high proportion of healthcare providers remained unprotected after HBV vaccination in Uzbekistan, especially hospital workers and physicians. Strategies to increase compliance with vaccination schedules are needed. Adoption of post-vaccination immunity testing policy can identify providers that may require revaccination.

**Disclosures:**

All Authors: No reported disclosures

